# Patient educational technologies and their use by patients diagnosed with localized prostate cancer

**DOI:** 10.1186/s12913-015-1090-y

**Published:** 2015-09-29

**Authors:** Richard J. Baverstock, R. Trafford Crump, Kevin V. Carlson

**Affiliations:** Faculty of Medicine, Division of Urology, University of Calgary and vesia [Alberta Bladder Centre], Calgary, AB Canada; Faculty of Medicine, Department of Surgery, University of Calgary, Calgary, AB Canada

**Keywords:** Health information technologies, Patient centered care, Patient education, Prostate cancer, Retrospective study

## Abstract

**Background:**

Two urology practices in Calgary, Canada use patient educational technology (PET) as a core component of their clinical practice. The purpose of this study was to determine how patients interact with PET designed to inform them about their treatment options for clinically localized prostate cancer.

**Methods:**

A PET library was developed with 15 unique prostate-related educational modules relating to diagnosis, treatment options, and potential side effects. The PET collected data regarding its use, and those data were used to conduct a retrospective analysis. Descriptive analyses were conducted and comparisons made between patients’ utilization of the PET library during first and subsequent access; Pearson’s Chi-Square was used to test for statistical significance, where appropriate.

**Results:**

Every patient (*n* = 394) diagnosed with localized prostate cancer was given access to the PET library using a unique identifier. Of those, 123 logged into the library and viewed at least one module and 94 patients logged into the library more than once. The average patient initially viewed modules pertaining to their diagnosis. Viewing behavior significantly changed in subsequent logins, moving towards modules pertaining to treatment options, decision making, and post-surgical information.

**Discussion:**

As observed through the longitudinal utilization of the PET library, information technology offers clinicians an opportunity to provide an interactive platform to meet patients’ dynamic educational needs. Understanding these needs will help inform the development of more useful PETs.

**Conclusion:**

The informational needs of patients diagnosed with clinically localized prostate cancer changed throughout the course of their diagnosis and treatment.

## Background

The provision of trustworthy information to patients and their families is a fundamental principle of providing patient-centered care [1]. It is also a core component of the informed consent process [2] and may increase patient safety and adherence [3]. Indeed, shared decision making, including patient education, has become a major policy initiative internationally in a variety of clinical settings and circumstances [4].

Patients diagnosed with localized prostate cancer are presented with several different types of therapies, each with known advantages and disadvantages [5]. Patients must make their treatment decision based on a careful weighing of the risks and benefits of each treatment option—but to make this choice, they need to be adequately informed.

Patient education has traditionally involved conversations between patients and their clinician, informational pamphlets, and/or audio- or video-tapes. However, there are limitations to these modes of communication. Previous research has observed that patients have a difficult time recalling important factual information provided by their health care provider [6], or often find that the conversation is dominated by their provider [7]. A two-part qualitative study aimed at improving doctor-patient communication found that patients do not fully voice concerns or ask questions about their diagnosis or treatment [8, 9]. Pamphlets and audio/video-tapes are linear and non-interactive [10].

Developments in information technology have moved patient education onto new platforms. Web-based platforms, phone applications, and social media have all been developed in the recent past to assist patients in better understanding their health condition. These “patient education technologies” (PETs) offer engaging material through interaction and can provide “just-in-time” learning that allows patients to access the information at their own pace and at a time that is best for them [11].

A systematic review of the use of internet and computer-based programs for prostate cancer patients found the use of these programs resulted in patient empowerment and an increased sense of control over their disease [11]. The authors also cited the advantage of computer programs as they can be easily updated with new treatment options [11].

This also supports recommendations that encourage clinicians and health educators to provide patients with only the information they need for decision making—patients using the PET may select the educational modules they are most interested in depending on the point of care [5, 12–14]. Web-based platforms also allow clinicians and health educators to track the most visited portions of the PET so that they can understand the informational needs of their patients.

Little is known about how patients interact or use PETs, particularly on a longitudinal basis. By filling this knowledge gap, clinicians would be better informed about the types of patients that engage with, and benefit from, these PETs. It would also provide these clinicians with an assessment of the kinds of information that their patients are accessing at different times throughout their care. Clinicians could use such information to make better use of their clinical visits with their patients, providing more supportive and more meaningful interactions.

Two urology practices in Calgary, Canada (KC & RB) currently use a PET library [15] as a core component of their clinical practice. This PET library is accessible via the internet, and includes a number of different modules pertaining to various aspects of urologic care. This PET library has been widely used for all patients newly diagnosed with clinically localized prostate cancer (<cT3 disease) since 2006. All patients are given a unique log-in code for accessing the PET library. This affords a unique opportunity to track patients’ utilization of the PET library over the course of their diagnosis and treatment.

Thus, the purpose of this study is to better understand how patients interact with a PET library aimed at informing them about clinically localized prostate cancer and its associated treatment options.

## Methods

Upon being diagnosed with localized prostate cancer, patients are “prescribed” access to the PET library. Non-English language patients or patients with cognitive deficits are still offered access to the PET library on the assumption that a friend of family member could assist the patient in reviewing the materials. The library is freely available online, using any internet-capable computer. Patients log into the library using the access credentials provided by their physician as part of the prescription. These login credentials are unique to each patient. Each patient is provided with a handout containing the login instructions.

Once logged in, patients choose from a list of modules relating to prostate cancer and its various treatment options (modules are described in detail below). Patients may select as many modules as they desire and may return to the library, using the same login credentials, as often as needed.

### Modules

**Fig. 1:**
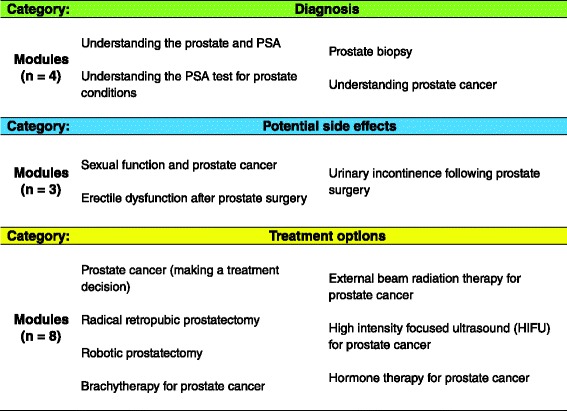
Modules within each category of the patient educational technology (PET) library for patients diagnosed with localized prostate cancer

The modules contained in the PET library were developed by an independent committee of urologists and based on systematic reviews of the literature; these are regularly updated as development in the evidence warrants. The PET library currently contains 15 different modules, which generally fall into one of three different categories: 1) information relating to diagnosis; 2) information relating to potential side effects; and 3) information relating to treatment options (see Fig. 1 for a full list of the modules). The PET was reviewed and approved by the Patient Information Committee of the Canadian Urological Association in 2007 and 2009 and has been accredited by the Health on the Net (HON) Foundation, which assesses the quality of health information available online [16].

Each module offers an animated presentation with narration. The module itself is comprised of a viewing area; play/stop/rewind/fast forward buttons, allowing the patient to control the tempo of the presentation; and a table of contents, which can be used to jump to specific topics of interest in the module’s presentation. The script for the narration is written at a grade ten reading level.

The software platform upon which the PET is built is linked to a database that stores data pertaining to a number of different utilization variables. The most relevant variables to this study include: unique identifier; amount of time the PET library was accessed; module(s) viewed during access; sequence of module viewership; play time for module(s); and the sequence of slides viewed in each module.

### Analysis

This study retrospectively analyzed access to the PET library between July 2006 and March 2011 (approximately 57 months). Access data was matched using patients’ unique login credentials to track their sessions longitudinally. This was done in order to identify and characterize patients’ utilization patterns. Modules that were viewed for less than 2 min were excluded from the analysis. This arbitrary trim point was made to differentiate between those patients who viewed the module, versus those who were just “browsing”. Descriptive analyses were conducted and comparisons made between patients’ utilization of the PET library during first and subsequent access; Pearson’s Chi-Square was used to test for statistical significance, where appropriate.

Prior to accessing the PET library, patients are required to review a user agreement/consent outlining the data that the software collects, how these data are secured, and how they may be used. Patients cannot advance to view the PET library before first agreeing with the terms of the agreement/consent. This study has been approved by the University of Calgary Bioethics Review Committee.

## Results

The PET’s database included observations from 399 access points. When aggregated to the patient-level unit of observation, this equated to 123 unique patients who had viewed at least one module for at least 2 min; 94 of these patients logged in more than once. Over the course of the study period, 394 patients had been referred to the PET library (i.e., diagnosed with localized prostate cancer) for a participation rate of 31 %. The average age of those who accessed the PET library was 56.4 years (range: 24–82 years). These patients viewed 3 modules on average (range: 1–12 modules; 25th percentile = 1 module, 75th percentile = 4 modules).

The average patient spent approximately 10 min viewing modules upon first accessing the PET library. This grew significantly (*p* < 0.025) to an average of 14 min in subsequent visits (range: 2–199 min). On average, patients returned to the PET for 43 days, ranging from 1–352 days (25th percentile = 1 day; 75th percentile = 51 days).

### Categories

**Fig. 2:**
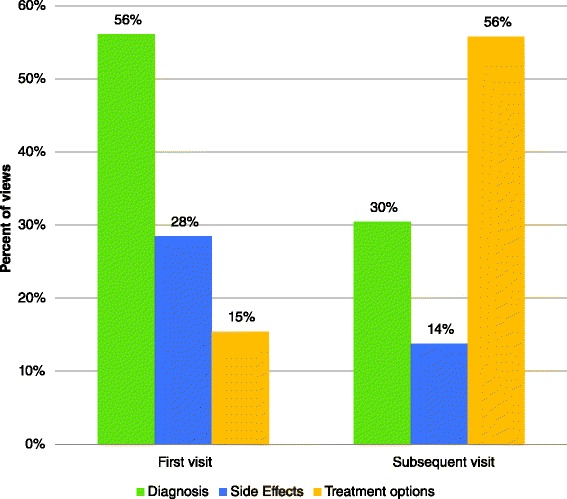
Information categories sought by patients diagnosed with localized prostate cancer using patient education technologies (PET) for prostate cancer education during first and subsequent visits to the PET website (*n* = 123, Calgary, Alberta, Canada)

Figure 2 describes the popularity of given categories of information between participants’ first and subsequent visits. Participants’ first visits were dominated by an interest in information relating to their diagnosis; 56 % of first modules viewed fell into this category. Information pertaining to potential side effects was the next most popular category (28 % of all initial visits viewed a module in this category), and modules relating to treatment options were the least popular (15 %).

Upon returning to the PET, participants’ interests changed. Those modules providing information on treatment options became the most popular, with 56 % of participants seeking this category of module in their subsequent visit. This was followed by 30 % of participants viewing modules relating to their diagnosis, and 14 % viewing modules on the potential side effects. The shift in interests from first to subsequent visits was statistically significant (Chi-square = 94.63; *p* < 0.01).

### Modules

**Fig. 3:**
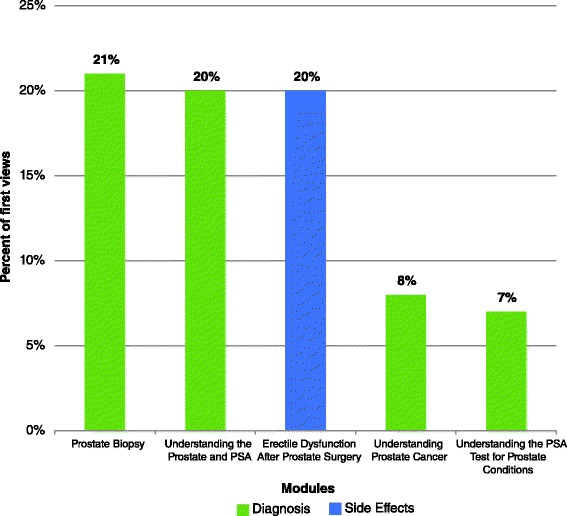
Five most frequently viewed modules by patients diagnosed with localized prostate cancer using patient education technologies (PET) for prostate cancer education during the patient’s first visit to the PET library (*n* = 123, Calgary, Alberta, Canada)

**Fig. 4:**
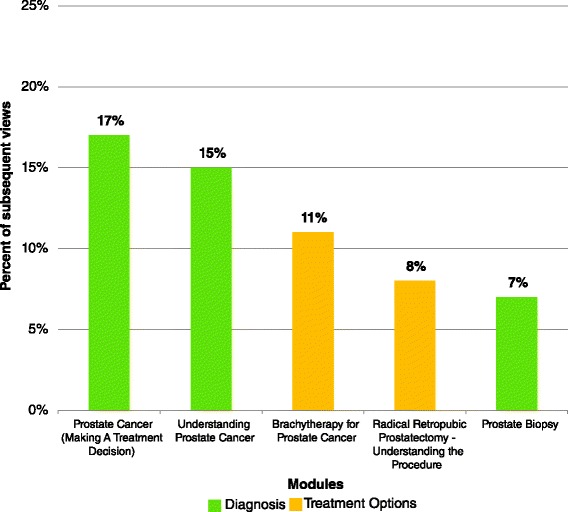
Five most frequently viewed modules by patients diagnosed with localized prostate cancer using patient education technologies (PET) for prostate cancer education during the patient’s subsequent visits to the patient education technologies (PET) library (*n* = 276, Calgary, Alberta, Canada)

Three modules in particular accounted for most of the interest at initial visit: 21 % of all initial visits viewed the module “prostate biopsy”, 20 % viewed the module “understanding the prostate and PSA” and 20 % viewed the module “erectile dysfunction after prostate surgery.” At subsequent visit, 17 % of all subsequent visits viewed the module “prostate cancer (making a treatment decision), 15 % viewed the module “understanding prostate cancer” and 11 % viewed the module “brachytherapy for prostate cancer.” Figures 3 and 4 illustrate the five most frequently viewed modules for participants’ first and subsequent visits, respectively.

### Information

Nearly three-quarters (74 %) of slides viewed during the initial visit were focused on clinical (e.g., “what is a normal PSA value?”), procedural (e.g., “preparing for the biopsy”), and anatomy/physiology (e.g., “biological function of the prostate”) information. Other issues of interest include information on potential risks (13 %) and side effects (13 %). During subsequent visits, 71 % of slides viewed include clinical, procedural, decision making (e.g., “understanding prostate cancer ‘risk’”) information. Participants’ broadened their interests, not only including information on potential risks and side effects, but also information on post-surgical care.

## Discussion

PETs offer an opportunity to better understand how patients’ patterns of information needs change after diagnosis with prostate cancer. The PET library used in this study provides far more insight into the informational needs of patients diagnosed with early stage prostate cancer than would a pamphlet or audio/video tape. Moreover, utilization data–similar to what is presented here, although on an individual basis–can be linked to the patient’s electronic medical record (EMR) allowing the clinician to follow-up with specific questions.

In this study we observed that only one third of patients accessed the PET library. Given the retrospective nature of this study and the restrictions placed on it by the ethics review board, we were limited in our ability to better understand why and who were accessing the PET and how they differed from those that did not. A future prospective study, with appropriate consent, is needed to investigate this important issue. Other similar studies of the use of web-based prostate cancer education tools found that these programs worked best with men less than 60 years of age and with a higher education level and better socioeconomic background [17, 18]. Our future research will strive to determine if these findings are consistent as the population ages and older adults, or those from diverse backgrounds, gain increased familiarity and access to internet-enabled computers.

Those who did access the PET library, though, did so multiple times. Participants chose to access the library over the course of several days and months, for different lengths of time. The modules of interest changed upon these subsequent visits, moving from modules relating to diagnosis to modules relating to treatment options. This is a logical transition, likely reflecting patients’ own navigation from diagnosis to treatment process, and is consistent with findings in other studies involving patient use of prostate cancer educational tools [11]. This was also reflected in the specific information they sought; from clinical and anatomical information to more procedural information and other issues (e.g., decision making and post-surgical concerns).

### Limitations

There are, however some limitations to this study that should be noted. First, the small sample of practice settings and patient population limit the generalizability of the results. A larger patient sample, drawn from more urologic practices, may have interacted with the PET library differently from that observed here. Our sample may also suffer from a self-selection bias that we are unable to detect here because of the small size and retrospective nature of the study design.

We are unable to identify whether it was the patient that accessed the PET library or a surrogate. This may skew observations for either the modules viewed or the information sought in an unknown direction. We do not, however, feel as though this detracts from the overall results. Surrogate involvement (e.g., spouse) is often encouraged as part of high quality patient-centered care [19], and their involvement of the PET library would accurately reflect how these technologies are used and shared by patients.

The study design and limitations based on ethics approvals does not allow us to present information on the differences between patients who accessed the PET library versus those who did not. It also did not allow us to link PET library use with other outcomes that may support its value, such as quality of life, clinical outcomes, or patient satisfaction.

## Conclusion

The informational needs of patients diagnosed with clinically localized prostate cancer changed throughout the course of their diagnosis and treatment, as observed through their first and subsequent utilization of an online PET library. To fulfill these needs those patients choosing to utilize a PET do so multiple times, seeking out different aspects and information at different times.

### Practical implications

This study provides an opportunity to observe the implementation of a PET in an uncontrolled environment (i.e., an active clinical urological setting). There were not strict inclusion or exclusion criteria, and there were no artificial financial incentives provided to patients or clinicians. All patients were given access to the PET library along with other forms of education (print material and a live group session) in a balanced manner. The observations here reflect the genuine interest and use of PETs for early stage prostate cancer. This can be useful to future clinicians and researchers interested in incorporating PETs into their own practices or in developing their own PETs.

Indeed there is much work that needs to be done in this area. With more patients seeking clinical information from online sources, there is a greater need to understand how patients use these types of information sources. Questions remain as to whether patients who accessed the PET library had more satisfaction with their treatment choice, higher quality of life, or better clinical outcomes. Future research directions should include an ability to link information on PET library use, scores on measures of quality of life, and clinical outcomes. Patients could also be engaged in a qualitative evaluation to understand how they feel about the PET library.

Patient education technologies may not only be for shared decision making, but also included in other areas of patient-centric programs, such as patient-specific guidelines [20] or interactive games [21]. Clinicians also need to develop a skill set in how to use this information as part of the two-way communication process with their patients. Moreover, patients are becoming more accepting of information technology in the health care setting and recognize the potential of this technology to better inform patients about their health and health care [22].

## Abbreviations

PET: Patient Educational Technology
HON: Health on Net
PSA: Prostate-specific Antigen
EMR: Electronic Medical Record
